# Inhibition of Oxidative Stress and Related Signaling Pathways in Neuroprotection

**DOI:** 10.3390/antiox13091033

**Published:** 2024-08-26

**Authors:** Maja Jazvinšćak Jembrek

**Affiliations:** 1 Division of Molecular Medicine, Ruđer Bošković Institute, Bijenička 54, 10000 Zagreb, Croatia; maja.jazvinscak.jembrek@irb.hr or maja.jazvinscak.jembrek@unicath.hr; 2 School of Medicine, Catholic University of Croatia, Ilica 242, 10000 Zagreb, Croatia

Oxidative stress, characterized by increased production of reactive oxygen species (ROS) and disturbed redox homeostasis, is one of the key mechanisms underlying synaptic loss and neuronal death in various neurodegenerative diseases [1]. It is closely linked to other pathological processes that exacerbate neuronal damage at both cellular and molecular levels. These includes disturbance of calcium homeostasis, endoplasmic reticulum stress, excitotoxicity, alterations of the brain lipid profile, impairment of mitochondrial function, and deregulation of intracellular signaling pathways [2]. Together with the formation of protein aggregates and aberrant protein clearance, these events initiate pronounced microglial activation, inflammatory response, and cytokine release, ultimately leading to cell death [3]. Recently, a great interest has been directed towards the ROS-induced nod-like receptor protein-3 (NLRP3) inflammasome, which promotes the release of inflammatory mediators and triggers pyroptosis, a form of inflammation-dependent programmed cell death that plays a critical role in neurodegenerative diseases [4,5]. Eventually, all these mechanisms compromise synaptic transmission and impair neuronal circuitry in specific brain areas, leading to the progressive decline of motoric and cognitive abilities [6].

As previously emphasized, the disruption of redox-regulated signaling cascades, which may occur in neurons, glia, and endothelial cells, plays an important role in triggering cell death and neuroinflammation in neurodegenerative diseases [7,8,9]. On the other hand, growing evidence suggests that re-tuning the activity of these pathways offers significant pharmacological possibilities for improving therapeutic strategies against neurodegeneration. Targeting these pathways with neuroprotective agents could enhance current therapeutic options, which are still very limited and often unsatisfactory [10,11].

The activation of transcription factors nuclear factor erythroid 2-related factor 2 (Nrf2) and nuclear factor kappa B (NF-κB) plays an essential and interacting role in tuning redox balance and inflammatory responses. These pathways have been identified as important targets for pharmacological interventions aimed at mitigating neuropathological changes [12,13,14]. However, it should be emphasized that a systematic review of human studies that measured Nrf2 activation has provided only limited evidence that phytochemicals may significantly induce Nrf2 activity. Therefore, controlled clinical trials are urgently needed to validate the promising findings from in vitro and animal studies regarding Nrf2-induction-based approaches in humans, at least in the context of dietary interventions [15]. In addition to Nrf2 and NF-κB, the aberrant activation of signaling pathways of mitogen-activated protein (MAP) kinases, including extracellular signal-regulated kinases (ERKs), c-Jun N-terminal kinases (JNKs), and p38 kinases, together with the disturbed activation of the Akt pathway, may also play an important role in neuronal toxicity and progressive neurodegeneration. Modulating the activity of these pathways could also have significant therapeutic implications [16,17,18].

The Special Issue “*Inhibition of Oxidative Stress and Related Signaling Pathways in Neuroprotection*” includes eight original research articles and one review paper, each addressing some specific aspect of neuroprotection. These studies explore the molecular and cellular mechanisms that contribute to neuroprotection and highlight potential therapeutic avenues. The focus is on recently discovered or rediscovered neuroprotective compounds that target signaling pathways and key cellular events associated with oxidative stress and neuroinflammation.

*1.1. Targeting the ROS/NLRP3/pyroptosis signaling pathway: effects of phytochemicals*

Bioactive molecules of natural origin are highly appreciated as a promising therapeutic option due to their favorable safety profile and multi-target mechanisms of action. These compounds are capable of mitigating neuronal injury by suppressing mitochondrial oxidative stress and neuroinflammatory responses [19]. Epimedium, a traditional Chinese herbal medicine, possesses powerful anti-inflammatory and antioxidant properties. A study by Wu and colleagues demonstrated that the primary active compounds in Epimedium, *icariin*, *icariside II*, and *icaritin*, directly interact with NLRP3, inhibiting the NLRP3 signaling cascade [20]. Inhibition of the NLRP3 inflammasome has been shown to reduce inflammatory responses, protein aggregation, and behavioral changes in animal models of neurodegenerative diseases [21,22]. Thus, regulating the NLRP3 inflammasome and ROS-mediated pyroptosis could be an effective strategy for developing novel therapies, although the safety and effectiveness of NLRP3 inhibitors have yet to be confirmed in clinical trials [23].

*1.2. New approaches against leukocyte infiltration and secondary inflammation without blood–brain barrier (BBB) breakdown: evidence from status epilepticus*

Status epilepticus is a severe medical condition characterized by an inflammatory response, infiltration of inflammatory cells, cytokine release, and neurodegeneration [24]. Various inflammatory mediators increase neuronal excitability by promoting the activation of astrocytes and microglia, as well as the expression of inflammatory genes [25]. Monocyte chemotactic protein-1 (MCP-1) and macrophage inflammatory protein-2 (MIP-2) regulate the infiltration of leukocytes into the brain parenchyma independent of BBB permeability. Therefore, targeting the expression of these chemokines and their receptors in microglial cells, astrocytes, and neurons, particularly in the frontoparietal cortex, offers a promising and innovative therapeutic approach for treating status epilepticus [26]. Furthermore, it has been shown that epigallocatechin-3-gallate (EGCG), the major polyphenolic compound in green tea, can reduce infiltration of immune cells. This effect is attributed to EGCG’s ability to modulate the activity of p38, ERK1/2, and NF-κB pathways, interact with multiple surface receptors and transcription factors, and prevent the induction of molecules that promote leukocyte recruitment [27]. However, further research is needed to fully characterize the therapeutic potential of EGCG, as well as other phytochemicals, in controlling inflammation, particularly regarding adverse effects, dosing regimens, and improved delivery options [28].

*1.3. Peroxiredoxin 6 (Prdx6) and clasmatodendrosis: insights from the hippocampus of rats with chronic epilepsy*

Clasmatodendrosis, a form of autophagy-mediated astroglial degeneration, has an important role in the duration of spontaneous seizures in rats with chronic epilepsy. This condition can be alleviated by N-acetylcysteine (NAC), a glutathione (GSH) precursor. NAC ameliorates clasmatodendrosis by increasing expression of glutamine synthase (GS) and GSH peroxidase 1 (GPx1), and by preventing the upregulation of peroxiredoxin 6 (Prdx6), a multifunctional enzyme that acts as an acidic calcium-independent phospholipase (aiPLA2) and GPx in astrocytes. These findings suggest that the ROS-Prdx6-GPx1-GS axis is crucial for plasmatodendritic degeneration. Specifically, it is likely that the aiPLA2 activity of Prdx6 abolishes the GPx1-mediated function of GS, impairing the glutamate–glutamine conversion and leading to degeneration of hippocampal astrocytes, thereby extending the duration of spontaneous seizure [29]. Altered Prdx6 activity has been associated with various CNS disorders, though its role appears to vary across different diseases, ranging from protective to damaging effects. Therefore, further research is necessary to better understand if Prdx6 could be a reliable target for therapeutic interventions in epilepsy and other neurodegenerative conditions [30].

*1.4. Pyruvate kinase M2 (PKM2) and cerebral ischemia-induced neuronal death*

PKM2 is a multifunctional enzyme that catalyzes the conversion of phosphoenolpyruvate to pyruvate during aerobic glycolysis. In astrocytes, pyruvate is further metabolized into lactate, which is then supplied to neurons. This suggests that PKM2 may play a key role in astrocyte-guided neuronal response to damage, particularly in the context of cerebral ischemia [31]. Accordingly, deletion of the *PKM2* gene has been shown to exacerbate neuronal damage and impair lactate metabolism in the hippocampus, highlighting the critical role of the astrocyte–neuron lactate shuttle [31]. This also points to the potential of lactate administration as a strategy to prevent oxidative stress, neuronal damage, and cognitive impairment following ischemic injury. Interestingly, the tetrameric form of PKM2 functions as a glycolytic enzyme in the cytosol, while the dimeric form acts as a transcriptional coactivator and protein kinase in the nucleus, regulating the expression of proteins involved in main signaling pathways and redox homeostasis [32]. There is substantial evidence that PKM2 has an important role in various inflammatory conditions. For example, nuclear PKM2 levels are elevated in neutrophils, driving a thrombo-inflammatory reaction during the pathogenesis of acute ischemic stroke [33]. Similarly, microglial PKM2 mediates neuroinflammation and neuronal loss in the hippocampus of mice with pilocarpine-induced status epilepticus. PKM2 activates microglia by upregulating the expression and phosphorylation of NF-κB, leading to the production of several inflammatory mediators, such as C1q, TNF-α, and IL-1α. These mediators, in turn, contribute to excessive C3 expression in astrocytes, which binds to neuronal C3a receptors, inducing neuronal damage [34]. Given these findings, further studies are needed to fully understand the therapeutic potential and possible adverse effects of targeting PKM2 in neuroinflammatory conditions.

*1.5. Dietary polyphenols at the crossroad of chemotherapy and neuronal damage in peripheral neuropathy*

Chemotherapy treatment often leads to neurotoxicity, neuroinflammation, and neurodegeneration, which can result in long-lasting neuropathic pain. This pain profoundly affects the quality of life of oncology patients and places a substantial burden on the healthcare system [35]. Chemotherapy-induced neuropathic pain can be challenging to manage in certain patients, often requiring a combination of pharmacologic and non-pharmacologic approaches. The antinociceptive effects of various phytochemicals have been explored in animal models of neuropathic pain. In this context, EGCG, along with other flavonoids and polyphenolic compounds, has emerged as a promising dietary intervention for reducing the pain threshold. This potential is attributed not only to their antinociceptive properties but also to their anti-inflammatory effects, a reduction in inflammatory pain through the prevention of immune cell infiltration, antioxidant, antiapoptotic, and neuroprotective abilities, and favorable safety profiles [36,37]. Based on these preclinical findings and positive effects of green tea extract in patients with diabetic peripheral neuropathy [38], it is essential to further evaluate the efficacy and safety of natural compounds in clinical studies for chemotherapy-induced neuropathic pain.

*1.6. Dietary polyphenols in alleviating symptoms* of *Parkinson’s disease (PD): a new perspective on Sambucus nigra flowers*

*Sambucus nigra* flowers (elderflowers) have long been used in traditional medicine for alleviating symptoms of the common cold. The recognized biological and pharmacological activities of *S. nigra* are attributed to the high content of polyphenols, such as anthocyanins and hydroxycinnamic acid, and triterpenes. However, recent evidence suggests that elderflower extracts may be beneficial in slowing the progression of neurodegenerative diseases based on the ability to strengthen antioxidant capacity and reduce ROS levels, inhibit the mammalian target of the rapamycin complex 1 (mTORC1) pathway, and restore autophagy. Additionally, the extracts may improve mitochondrial function and promote the clearance of pathological protein aggregates and α-synuclein oligomerization in vitro [39,40]. Animal studies further demonstrate that bioactive compounds from *Sambucus* plants can improve cognitive and motor performances as well as neuronal survival. These benefits are attributed to their ability to attenuate oxidative stress and neuroinflammation, modulate signaling pathways, reduce apoptosis, and enhance mitochondrial function. Hence, incorporating the bioactive compounds derived from *Sambucus* plants into functional food could be an innovative approach for promoting brain health and cognitive functions, but further research is needed to address the stability, bioavailability, and organoleptic properties of such dietary supplements, as well as their safety and efficacy in humans [41].

*1.7. Drug repurposing: omarigliptin as a new hope for alleviating symptoms of* *PD*

Omarigliptin, a dipeptidyl peptidase-4 inhibitor, is a drug used to treat type 2 diabetes [42]. It increases the levels of incretin hormones, such as glucagon-like peptide-1 (GLP-1) and glucose-dependent insulinotropic polypeptide (GIP), which help to increase blood glucose levels. Recent research suggests that omarigliptin may also be repurposed for treating neurodegenerative diseases. Docking studies indicate that omarigliptin can bind to A_2A_ adenosine receptor which regulates glutamate and dopamine release in PD, as well as to acetylcholine esterase (AChE) receptors. Additionally, omarigliptin increases brain GLP-1 levels and effectively crosses the BBB when applied in multiple doses [43]. Omarigliptin also demonstrated antioxidative, anti-inflammatory, and neuroprotective effects against rotenone- and 6-hydroxydopamine-induced toxicity in PC12 cells. These effects are likely due to its DPP-4 inhibitory activity and increased GLP-1 levels, which may help in counteracting the harmful effects of neurotoxic compounds [44]. Furthermore, omarigliptin reduced ROS production and the extent of lipid peroxidation by upregulating Nrf2 and its target protein heme-oxygenase 1 (HO-1). It also exhibited prominent anti-inflammatory effects through the inhibition of NF-κB activation in an Akt-dependent manner, along with antiapoptotic properties [45]. Taken together, these findings highlight the therapeutic potential of omarigliptin and other DPP-4 inhibitors as therapeutic agents for neurodegenerative conditions.

*1.8. Challenges of animal models and discovery of novel neuroprotective compounds for neurodegenerative diseases*

In vitro and animal models are crucial for studying neurodegenerative diseases and discovering novel neuroprotective agents. Reliable animal models should provide meaningful insights into disease mechanisms and potential treatments while being relevant to human diseases in the context of genetic similarity, replication of key pathological features (including behavioral and cognitive deficits), disease progression, and drug response. Despite their importance, animal models come with certain challenges. The common limitations of animal models include genetic and physiological differences and incomplete recapitulation of disease pathology compared to humans, lack of genetic diversity, behavioral and cognitive differences, and costs of care and handling, among others [46]. All these may compromise the utility and translatability of research findings to human therapies. Alarmingly, the failure rate of the translating findings from animal studies to human treatments exceeds 92%, primarily due to unforeseen toxicity and lack of efficacy [47]. In accordance with the 3Rs principles to replace, refine, and reduce mammalian species in various studies, and in response to aforementioned challenges, research efforts are also focused on developing novel model organisms and human-relevant research methodologies, particularly for identifying novel therapeutics and their neurotoxicological evaluation at the biochemical and behavioral level [48]. However, some researchers argue against using models that only partially mimic the phenotype of human diseases, questioning their value for discovering therapeutically relevant compounds for humans [46].

*1.9. The endocannabinoid system as a target for treating neurodegenerative diseases*

Recent studies have highlighted the neuroprotective potential of endocannabinoids, plant-derived cannabinoids, and synthetic cannabinoids against the most common neurodegenerative conditions [49]. Although cannabinoids display a range of beneficial effects, such as antioxidative, anti-inflammatory, and antiapoptotic activities, the mechanisms behind their actions are rather complex and not yet fully understood due to the numerous intracellular targets and downstream effectors. Cannabinoids interact with CB1 receptors to activate various signaling pathways involved in oxidative defense and cell survival, while through CB2 receptors, they can suppress the release of pro-oxidative and pro-inflammatory mediators. Additionally, cannabinoids may also engage with other receptors [50]. Therefore, their potential as a research avenue in alleviating neurodegeneration warrants further investigation. Future studies should focus on elucidating the interactions between activated receptors, signaling pathways, and molecular targets, as well as assessing the safety profile and potential adverse effects associated with long-term use [51,52].

In conclusion, while the findings presented in this Special Issue are promising, the therapeutic potential of discussed compounds and targeted mechanisms requires further validation. This validation should occur not only in animal models, despite their limitations, but also, and more critically, in clinical trials. Ultimately, the results highlighted here have the potential to advance the development of more effective pharmacological strategies against neuropathological mechanisms underlying neurodegenerative changes in various conditions.
